# Serum KLF2, YKL-40, and vitamin A levels and their prognostic value in neonatal respiratory distress syndrome

**DOI:** 10.3389/fped.2026.1898953

**Published:** 2026-10-29

**Authors:** Na Li, Feifei Yuan

**Affiliations:** 1 Department of Neonatology, Shanxi Children’s Hospital & Shanxi Women and Children’s Healthcare Hospital, Taiyuan, Shanxi, China; 2 Department of Neonatology, Guangzhou Key Laboratory of Neonatal Intestinal Diseases, The Third Affiliated Hospital, Guangzhou Medical University, Guangzhou, Guangdong, China; 3 Guangdong Provincial Key Laboratory of Major Obstetric Diseases, Guangdong Provincial Clinical Research Center for Obstetrics and Gynecology, Guangdong-Hong Kong-Macao Greater Bay Area Higher Education Joint Laboratory of Maternal-Fetal Medicine, The Third Affiliated Hospital, Guangzhou Medical University, Guangzhou, Guangdong, China

**Keywords:** neonatal respiratory distress syndrome, prognostic value, serum KLF2, vitamin A, YKL-40

## Abstract

**Objective:**

This study aimed to clarify the clinical significance of serum Krüppel-like factor 2 (KLF2), chitinase-3-like protein 1 (YKL-40), and vitamin A levels in neonatal respiratory distress syndrome (NRDS) and to determine their utility in predicting adverse outcomes.

**Methods:**

This study included 120 neonates with NRDS and 60 healthy controls. NRDS cases were stratified by radiographic severity into mild, moderate, and severe subgroups, and further divided into survival and non-survival groups based on outcomes. Serum KLF2, YKL-40, and vitamin A levels were measured and compared across groups. Logistic regression analysis was used to identify factors influencing NRDS prognosis, and a nomogram model was constructed. Predictive performance was evaluated using ROC curves, calibration curves, and DCA.

**Results:**

Serum KLF2 and vitamin A concentrations progressively decreased with increasing disease severity, whereas YKL-40 levels showed the opposite trend (all *P* < 0.05). Compared with survivors, non-survivors exhibited markedly lower KLF2 and vitamin A levels but elevated YKL-40 levels (all *P* < 0.05). Multivariable analysis demonstrated that KLF2, YKL-40, and vitamin A were independently associated with adverse outcomes in NRDS. These factors were used to construct a nomogram prediction model with a total score of 160 points (corresponding to a probability of 0.16–0.8). The ROC curve showed an AUC of 0.918 (95% CI: 0.863–0.973); the calibration curve indicated good discrimination and consistency; and the DCA curve verified a positive net benefit within the predictive probability threshold range of 0–0.88.

**Conclusion:**

Alterations in serum KLF2, YKL-40, and vitamin A levels are closely linked to disease severity and clinical outcome in neonates with NRDS. Combined assessment of these biomarkers may offer a practical approach for early prognostic stratification and risk evaluation.

## Introduction

Neonatal respiratory distress syndrome (NRDS) remains one of the leading causes of neonatal morbidity and mortality worldwide (1). The disorder is primarily caused by insufficient pulmonary surfactant production, resulting in diffuse alveolar collapse, impaired gas exchange, and severe hypoxemia (2). Although advances in surfactant replacement therapy and respiratory support have significantly improved survival (3), severe NRDS continues to be associated with substantial mortality and long-term respiratory complications, particularly in resource-limited settings (4). Early identification of infants at high risk of disease progression or poor outcomes is therefore essential for optimizing treatment strategies and improving prognosis.

Krüppel-like factor 2 (KLF2) is a zinc-finger transcription factor involved in endothelial homeostasis, anti-inflammatory regulation, and vascular protection (5). Emerging evidence suggests that KLF2 also contributes to lung development and protects against pulmonary injury by modulating inflammatory and oxidative stress responses (6). Experimental studies have shown that reduced KLF2 expression is associated with aggravated lung injury and endothelial dysfunction (7). Chitinase-3-like protein 1 (YKL-40), an inflammatory glycoprotein secreted by activated macrophages and neutrophils, has been implicated in tissue remodeling and inflammatory lung diseases (8). Elevated YKL-40 levels have been reported in bronchopulmonary dysplasia and acute respiratory distress syndrome and are associated with disease severity and adverse clinical outcomes (9–11). However, evidence regarding the clinical significance of KLF2 and YKL-40 in NRDS remains limited. Vitamin A is essential for normal lung morphogenesis, alveolar maturation, and maintenance of epithelial integrity. Deficiency of vitamin A has long been recognized as a risk factor for NRDS and other neonatal pulmonary disorders (12). Inadequate vitamin A levels may impair alveolar repair and epithelial regeneration, thereby increasing susceptibility to respiratory complications (12). Unlike KLF2 and YKL-40, which mainly reflect endothelial and inflammatory alterations, vitamin A represents nutritional and developmental status. Together, these biomarkers may provide complementary information regarding the complex pathophysiological processes underlying NRDS.

Despite increasing evidence linking KLF2, YKL-40, and vitamin A to pulmonary injury and repair, their combined clinical significance in NRDS has not been systematically investigated. To our knowledge, no previous study has simultaneously evaluated these biomarkers in relation to both disease severity and prognosis in NRDS. Therefore, the present study aimed to evaluate serum KLF2, YKL-40, and vitamin A levels in NRDS infants with different disease severities and to assess their individual and combined value in evaluating disease severity and predicting mortality. The findings may contribute to improved risk stratification and clinical decision-making in the management of NRDS.

## Materials and methods

### Ethics statement

This study was approved by the Ethics Committee of Shanxi Children's Hospital & Shanxi Women and Children's Healthcare Hospital and written informed consent was obtained from the legal guardians of all participants. Since all participants are newborns, written informed consent was signed by their legal guardians.

### Subjects

From January 2022 to December 2023, 120 neonates diagnosed with NRDS at Shanxi Children's Hospital & Shanxi Women and Children's Healthcare Hospital were enrolled. Diagnosis was established according to the relevant diagnostic criteria for NRDS (13). Neonates were diagnosed through imaging examinations and clinical signs. Chest x-rays showed diffuse bilateral pulmonary opacities with pulmonary edema, and echocardiography revealed no features of left atrial hypertension. Eligible participants had no major congenital malformations or inherited disorders, and written informed consent was obtained from their legal guardians. Neonates with severe extrapulmonary infection, chromosomal abnormalities, transient tachypnea of the newborn, aspiration pneumonia, or incomplete clinical data were excluded.

Additionally, 60 neonates without NRDS who were born during the same period at our institution were included as controls.

### Treatment of NRDS

① Pulmonary surfactant therapy: Based on a comprehensive assessment of the patient's condition, family wishes, and family economic conditions, a decision was made whether to supplement exogenous pulmonary surfactant. The dosage was administered according to the treatment dose specified in the instructions for exogenous pulmonary surfactants (poractant alfa injection 200 mg/kg, bovine pulmonary phospholipid injection 140 mg/kg). The patient was placed in the supine position, and the drug was administered via endotracheal intubation. The airway was cleared before administration, and pulmonary surfactant was slowly injected into the airway after rewarming. Immediately after administration, positive pressure ventilation with a resuscitation bag was given for approximately 5 min to prevent drug reflux. Within 6 h after administration, sputum suction and airway clearance were not performed. During and after administration, ventilator parameters were adjusted promptly to prevent complications such as pulmonary air leak.

② General treatment: Keeping warm, intravenous nutrition, maintaining internal environment stability, and selecting an appropriate respiratory support mode according to the condition: nasal cannula oxygen inhalation, heated humidified high-flow nasal cannula ventilation, continuous positive airway pressure (CPAP), conventional mechanical ventilation (CMV), and high-frequency ventilation (HFV). Indications for CPAP: high-risk preterm infants who may develop respiratory distress syndrome (e.g., those with gestational age <30 weeks who do not require endotracheal intubation and mechanical ventilation); oxygen fraction >0.3 during oxygen inhalation via nasal cannula or mask, arterial partial pressure of oxygen <50 mmHg, or transcutaneous oxygen saturation <90%; apnea of prematurity; RDS patients whose condition is stable after pulmonary surfactant administration and after extubation; marked three-concave sign and/or respiratory distress after weaning from invasive ventilation. Indications for CMV: frequent apnea that is ineffective after drug or CPAP intervention; RDS patients who require pulmonary surfactant; oxygen fraction >0.6, oxygen partial pressure <50 mmHg, or transcutaneous oxygen saturation <85%; neonates under general anesthesia. HFV is often used as rescue therapy after CMV failure, and can also be used for pulmonary air leak syndrome, certain congenital diseases, and RDS in preterm infants.

### Clinical data of patient

Demographic and clinical characteristics were obtained from the electronic medical records of all participants. Maternal variables included age, gravidity, gestational hypertension, and gestational diabetes mellitus. Neonatal characteristics included gestational age, sex, birth weight, postnatal age, term or preterm status, premature rupture of membranes, and mode of delivery.

### Serum sample collection and laboratory measurements

For infants with NRDS, the peripheral venous blood was collected within 24 h after diagnosis. For controls, blood samples were obtained within 24 h after birth. Following centrifugation, serum samples were isolated and stored at −80 °C until analysis. Serum concentrations of KLF2 and YKL-40 were determined using commercially available ELISA kits. Absorbance was measured at 450 nm, and concentrations were calculated using standard calibration curves. The KLF2 ELISA kit was obtained from Reddot Biotech (China), whereas the YKL-40 ELISA kit was purchased from R&D Systems (USA). Serum vitamin A levels were quantified by high-performance liquid chromatography (HPLC). Chromatographic separation was performed on a C18 reversed-phase column (250 mm × 4.6 mm, 5 μm) using a methanol–water mobile phase (95:5, v/v) at a flow rate of 1.0 mL/min. Detection was employed at 325 nm with the column maintained at 25 °C, and the injection volume was 20 μL. Retinol standards were utilized to generate calibration curves, and vitamin A concentrations were calculated from the corresponding peak areas.

### Assessment of the severity of NRDS in affected neonates

Based on the initial chest radiographic findings, neonates with NRDS were classified into mild (*n* = 40), moderate (*n* = 40), and severe (*n* = 40) groups according to the grading criteria described in Practical Pediatric Radiology Diagnostics (14). Mild NRDS (grade I) was characterized by a slight reduction in bilateral lung transparency accompanied by diffuse fine reticulogranular or ground-glass opacities. Air bronchograms were absent or confined to the hilar region, and the cardiac and diaphragmatic borders remained clearly visible. Moderate NRDS (grades II–III) was characterized by a more pronounced decrease in lung transparency and aggravated ground-glass changes. Air bronchograms extended beyond the cardiac silhouette into the middle and peripheral lung fields, while the cardiac and diaphragmatic borders became partially obscured. Diffuse fine granular and reticular opacities were commonly observed throughout both lungs. Severe NRDS (grade IV) was characterized by markedly reduced bilateral lung transparency with extensive diffuse ground-glass opacification. Air bronchograms became more prominent and extended from the hilar region toward the peripheral airways. The cardiac and diaphragmatic silhouettes were nearly or completely obscured, and in the most severe cases, a characteristic “white lung” appearance was observed.

### Prognostic outcome assessment

With the confirmation of NRDS diagnosis in newborns as the starting point and cure/discharge or death as the study endpoints, patients were divided into a survival group (those cured or improved and discharged) and a death group (those who died).

### Sample size estimation

Sample size estimation was performed prior to the study based on the planned binary logistic regression analysis used to identify independent prognostic factors associated with mortality in neonates with NRDS. Serum KLF2, YKL-40, and vitamin A levels were considered the primary predictor variables. According to a previous study (15) and preliminary data obtained by our research group, the mortality rate among neonates with NRDS was estimated to be approximately 21% (25/120), and an odds ratio (OR) of 2.5 was considered clinically meaningful. The sample size was estimated based on the multifactor logistic regression formula proposed by Hsieh et al. (16): $n\;=\;\frac{{(Z_{1-\frac{\alpha }{2}}+Z_{1-\beta })}^{2}}{\rho_{1}(1-\rho_{1})(\mathrm{lnOR}{)}^{2}}$, with a significance level *α* = 0.05 (two-sided test) and a power of 1−*β* = 0.80, we obtained Z1−*α*/2 = 1.96 and Z1−*β* = 0.84. Thus, *ρ*_1_ (1−*ρ*_1_) = 0.21 × 0.79 = 0.1659, lnOR = ln(2.5) ≈ 0.9163, (lnOR)^2^  0.8396, substituting into the formula, $n\;=\;\frac{7.84}{0.1659\times 0.8396}\approx \;57$ cases. To compensate for possible missing data and loss to follow-up, the sample size was further increased by 20%, resulting in a target enrollment of at least 69 patients. A total of 120 neonates with NRDS were ultimately included in the study, which substantially exceeded the minimum sample size requirement.

### Statistical methods

Statistical processing was completed using SPSS version 25.0 and GraphPad Prism version 10.0. Data distribution was determined according to the results of the Shapiro–Wilk test. Variables exhibiting normality were summarized as mean ± standard deviation (SD). Comparisons involving three groups were examined using one-way analysis of variance (ANOVA), followed by LSD *post hoc* testing where appropriate, whereas differences between two groups were assessed with the independent-samples t-test. For variables that did not satisfy the assumption of normality, results were expressed as the median and interquartile range (IQR). The Kruskal–Wallis H test was adopted for multiple-group comparisons, while the Mann–Whitney U test was used for pairwise analyses. Categorical variables were summarized as frequencies and percentages (n, %), and between-group differences were examined using either the Chi-square (*χ*^2^) test or Fisher's exact test, depending on data characteristics. Potential factors associated with clinical outcomes in neonates with NRDS were explored using univariate and multivariate logistic regression analysis. Before multivariate regression analysis, tolerance and variance inflation factor were used to diagnose multicollinearity among influencing factors. A prediction model was established and a nomogram was drawn, with internal validation performed using cross-validation. Receiver operating characteristic (ROC) curves and calibration curves were drawn, and the area under the ROC curve (AUC) and calibration curve were calculated to evaluate the accuracy and stability of the model. Finally, decision curve analysis (DCA) was used to confirm the clinical utility of the model. A two-sided *P* value below 0.05 indicated statistical significance.

## Results

### Baseline characteristics

Baseline maternal and neonatal characteristics were comparable across the control, mild, moderate, and severe NRDS groups. No significant differences were identified with respect to maternal age, gravidity, gestational hypertension, gestational diabetes mellitus, gestational age at birth, neonatal sex, birth weight, postnatal age, term status, premature rupture of membranes, or cesarean delivery among the control, mild, moderate, and severe NRDS groups (*P* > 0.05). Further pairwise comparison analysis showed that there were no significant differences between the control group and the mild, moderate, and severe groups in maternal age, gravidity, gestational hypertension, gestational diabetes mellitus, gestational age at birth, neonatal sex, birth weight, postnatal age, term status, premature rupture of membranes, or cesarean delivery (*P* > 0.05); there were no significant differences between the mild group and the moderate and severe groups in maternal age, gravidity, gestational hypertension, gestational diabetes mellitus, gestational age at birth, neonatal sex, birth weight, postnatal age, term status, premature rupture of membranes, or cesarean delivery (*P* > 0.05); there were no significant differences between the moderate group and the severe group in maternal age, gravidity, gestational hypertension, gestational diabetes mellitus, gestational age at birth, neonatal sex, birth weight, postnatal age, term status, premature rupture of membranes, or cesarean delivery (*P* > 0.05) (Table 1).

**Table 1:** Comparison of general information among the four groups.

Item	Control group (*n* = 60)	Mild group (*n* = 40)	Moderate group (*n* = 40)	Severe group (*n* = 40)	F/H$‘\chi^{2}$/	*P*
Maternal age (years)	28.82 ± 3.82	29.38 ± 3.53	29.83 ± 4.01	29.15 ± 4.30	0.555	0.645
Gravidity (times)	2 (1, 2)	2 (1, 2)	2 (1, 2)	2 (1, 2)	1.241	0.743
Gestational hypertension (%)	30 (50.00%)	21 (52.50%)	21 (52.50%)	20 (50.00%)	0.111	0.990
Gestational diabetes mellitus (%)	28 (46.67%)	20 (50.00%)	18 (45.00%)	19 (47.50%)	0.212	0.976
Gestational age at birth (weeks)	34.37 ± 2.41	33.63 ± 3.19	33.43 ± 2.70	34.01 ± 3.25	1.044	0.374
Neonatal sex (male/female)	30/30	21/19	22/18	19/21	0.511	0.916
Birth weight (kg)	2.33 ± 0.92	2.58 ± 0.80	2.40±1.02	2.75 ± 0.89	2.024	0.112
Postnatal age (h)	28.67 ± 5.82	27.98 ± 6.12	28.45 ± 5.72	27.93 ± 5.62	0.185	0.906
Term or preterm status (%)	26 (43.33%)	18 (45.00%)	14 (35.00%)	16 (40.00%)	1.010	0.799
Premature rupture of membranes (%)	24 (40.00%)	16 (40.00%)	17 (42.50%)	16 (40.00%)	0.081	0.994
Cesarean section (%)	31 (51.67%)	19 (47.50%)	21 (52.50)	21 (52.50%)	0.278	0.964

### Serum biomarker profiles across NRDS severity groups

Relative to controls, infants with NRDS exhibited reduced KLF2 and vitamin A concentrations accompanied by elevated YKL-40 levels (*P* < 0.05). Compared with the mild group, KLF2 concentrations were decreased and YKL-40 concentrations were increased in the moderate and severe groups (*P* < 0.05); compared with the moderate group, KLF2 and vitamin A concentrations were decreased and YKL-40 concentrations were increased in the severe group (*P* < 0.05) (Table 2).

**Table 2:** Comparison of serum markers in children with NRDS of different severity ($\bar{x}\pm s$).

Markers	Control group (*n* = 60)	Mild group (*n* = 40)	Moderate group (*n* = 40)	Severe group (*n* = 40)	F	*P*
KLF2 (pg/ml)	2.07 ± 0.11	1.71 ± 0.25*	1.66 ± 0.26^*&^	1.43 ± 0.32^*&#^	64.119	<0.001
YKL-40 (mg/L)	49.98 ± 5.68	64.49 ± 12.04*	73.25 ± 10.95^*&^	80.43 ± 5.63^*&#^	112.334	<0.001
Vitamin A (μmol/L)	0.82 ± 0.23	0.60 ± 0.27*	0.59 ± 0.25*	0.49 ± 0.18^*#^	17.81	<0.001

*vs. control group, *P* < 0.05; & vs. mild group, *P* < 0.05; # vs. Moderate group, *P* < 0.05.

### Baseline characteristics according to clinical outcome

Comparison of demographic and perinatal variables revealed no significant differences between survivors and non-survivors. Maternal age, gravidity, pregnancy-related complications, gestational age at birth, neonatal sex, birth weight, postnatal age, term status, premature rupture of membranes, and delivery mode were similarly distributed between the two groups (all *P* > 0.05) (Table 3).

**Table 3:** Comparison of general characteristics of children with NRDS of different outcomes.

Item	Non-survivor group (*n* = 21)	Survivor group (*n* = 99)	t/χ²	*P*
Maternal age (years)	29.19 ± 3.71	29.51 ± 4.00	0.332	0.741
Gravidity (times)	2.00 (1.00, 2.00)	2.00 (1.00, 2.00)	0.604	0.546
Gestational hypertension (%)	14 (66.67)	48 (48.48)	2.293	0.130
Gestational diabetes mellitus (%)	9 (42.86)	48 (48.48)	0.220	0.629
Gestational age at birth (weeks)	33.94 ± 2.88	33.64 ± 3.08	0.409	0.683
Neonatal sex (male/female)	11/10	51/48	0.005	0.943
Birth weight (kg)	2.59 ± 0.97	2.58±0.90	0.105	0.916
Postnatal age (h)	29.10 ± 5.62	27.91 ± 5.82	0.853	0.395
Term or preterm status (%)	11 (52.38)	37 (37.37)	1.626	0.202
Premature rupture of membranes (%)	7 (33.33)	42 (42.42)	0.593	0.441
Cesarean section (%)	13 (61.90)	48 (48.48)	1.248	0.264

### Serum biomarkers according to outcome status

Distinct differences in biomarker profiles were evident between outcome groups. Compared with survivors, non-survivors exhibited lower circulating KLF2 and vitamin A levels, whereas serum YKL-40 concentrations were markedly elevated (*P* < 0.05) (Table 4).

**Table 4:** Comparison of serum markers between NRDS infants with different outcomes ($\bar{x}\pm s$).

Markers	Non-survivor group (*n* = 21)	Survivor group (*n* = 99)	t	*P*
KLF2 (pg/mL)	1.34 ± 0.30	1.66 ± 0.27	4.66	<0.001
YKL-40 (mg/L)	84.47 ± 7.84	70.23 ± 11.03	5.615	<0.001
Vitamin A (μmol/L)	0.42 ± 0.12	0.59 ± 0.25	3.112	0.002

### Logistic regression analysis

Univariate regression analysis and multivariate regression analysis were used to investigate predictive factors affecting the prognosis of neonates with NRDS. With NRDS neonate prognosis status (survival = 0, death = 1) as the dependent variable, the results of univariate regression analysis showed that serum KLF2, YKL-40, and vitamin A were associated with the prognosis of neonates with NRDS (*P* < 0.05). Variables with *P* < 0.05 in the univariate regression analysis results were included in the multivariate regression model. Prior to the analysis, a multicollinearity test was performed, which showed no severe collinearity among the variables (Supplementary Table S1). The multivariate logistic regression analysis revealed that serum KLF2 (OR = 0.099; 95% CI = 0.012–0.807, *P* = 0.031), YKL-40 (OR = 1.190; 95% CI = 1.075–1.317, *P* = 0.001), and vitamin A (OR = 0.020; 95% CI = 0.001–0.726, *P* = 0.033) were predictive factors for the prognosis of neonates with NRDS (Table 5).

**Table 5:** Logistic regression analysis of factors associated with prognosis in infants with NRDS.

Variables	Univariate regression analysis						Multivariate regression analysis					
	B	Standard Error	Wald	*P*	OR	95% CI	B	Standard Error	Wald	*P*	OR	95% CI
KLF2	−3.867	0.991	15.223	<0.001	0.021	0.003∼0.146	−2.317	1.073	4.663	0.031	0.099	0.012–0.807
YKL40	0.195	0.049	15.893	<0.001	1.216	1.104∼1.338	0.174	0.052	11.336	0.001	1.19	1.075–1.317
Vitamin A	−3.859	1.346	8.22	0.004	0.021	0.002∼0.295	−3.912	1.832	4.558	0.033	0.02	0.001–0.726
Constant	-	-	-	-	-	-	−9.895	4.512	4.809	0.028	-	-

### Predictive performance analysis

The prognostic performance of serum KLF2, YKL-40, and vitamin A was further examined using ROC curve analysis. The corresponding AUC values were 0.799, 0.848, and 0.673, respectively. When the three biomarkers were combined, discriminatory performance improved further, yielding an AUC of 0.918 with a sensitivity of 0.905 and a specificity of 0.859 (Table 6; Figure 1).

**Table 6:** Predictive performance for serum YKL-40, KLF2, and vitamin A for severe NRDS.

Test variable	AUC	Standard error	*P*	95% CI	Sensitivity	Specificity	Cut-off
KLF2	0.779	0.065	<0.001	0.652–0.906	0.667	0.869	1.020
YKL-40	0.848	0.041	<0.001	0.768–0.928	0.905	0.636	76.860
Vitamin A	0.731	0.049	0.001	0.636–0.826	0.952	0.525	0.630
Combined	0.918	0.028	<0.001	0.863–0.973	0.905	0.859	-

**Figure 1:**
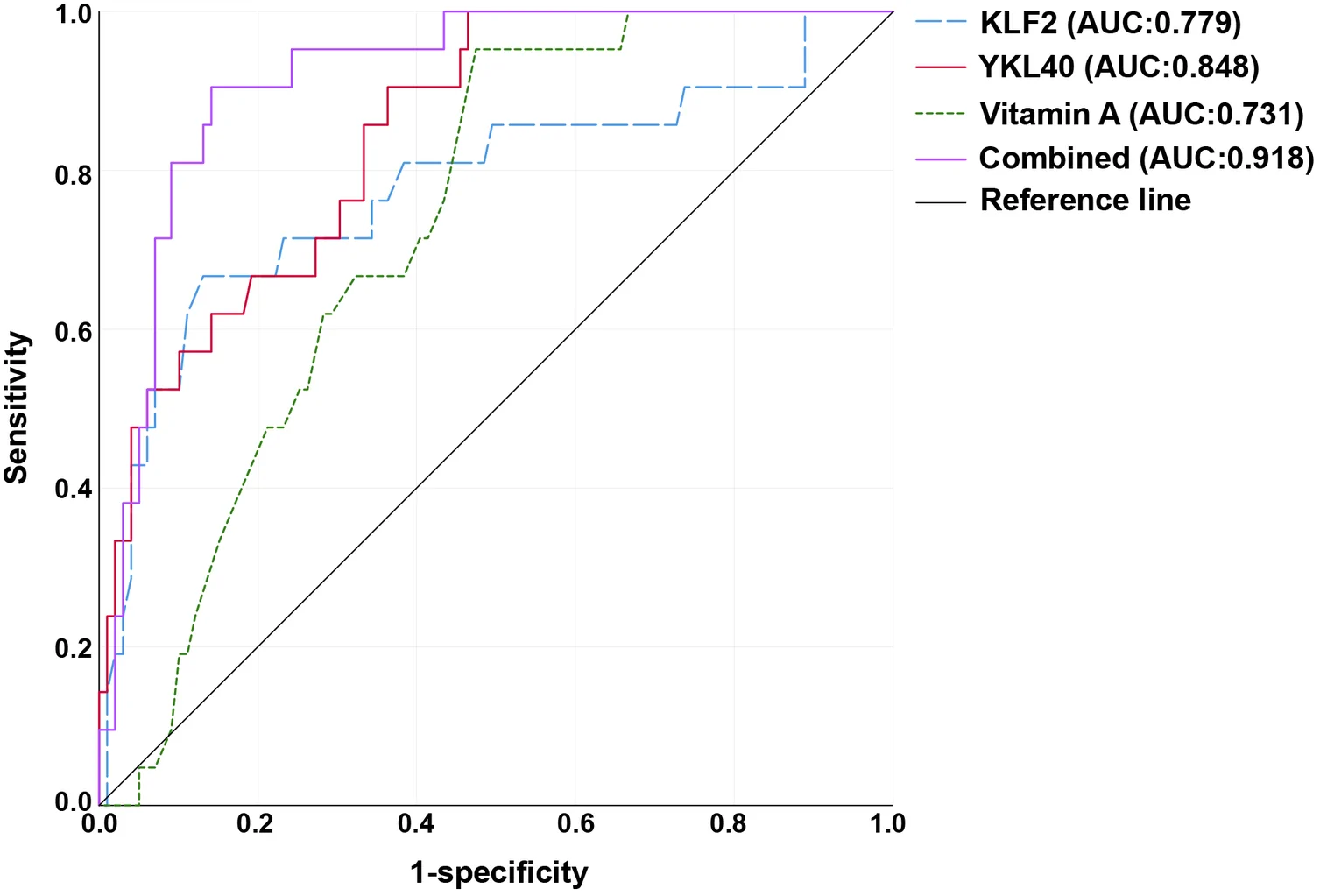
ROC curve of serum KLF2, YKL-40, and vitamin A in predicting prognosis and mortality in NRDS.

### Construction of the nomogram model

Based on the results of Logistic regression analysis of factors affecting the prognosis of neonates with NRDS, the three variables KLF2, YKL-40, and vitamin A were used as predictors to construct a nomogram model for the risk of poor prognosis in neonates with NRDS (Figure 2). KLF2, YKL-40, and vitamin A were each assigned a score, with a total score of 0–160 points corresponding to a predicted probability of 0.16–0.80. The higher the patient's score, the higher the risk of poor prognosis in neonates with NRDS.

**Figure 2:**
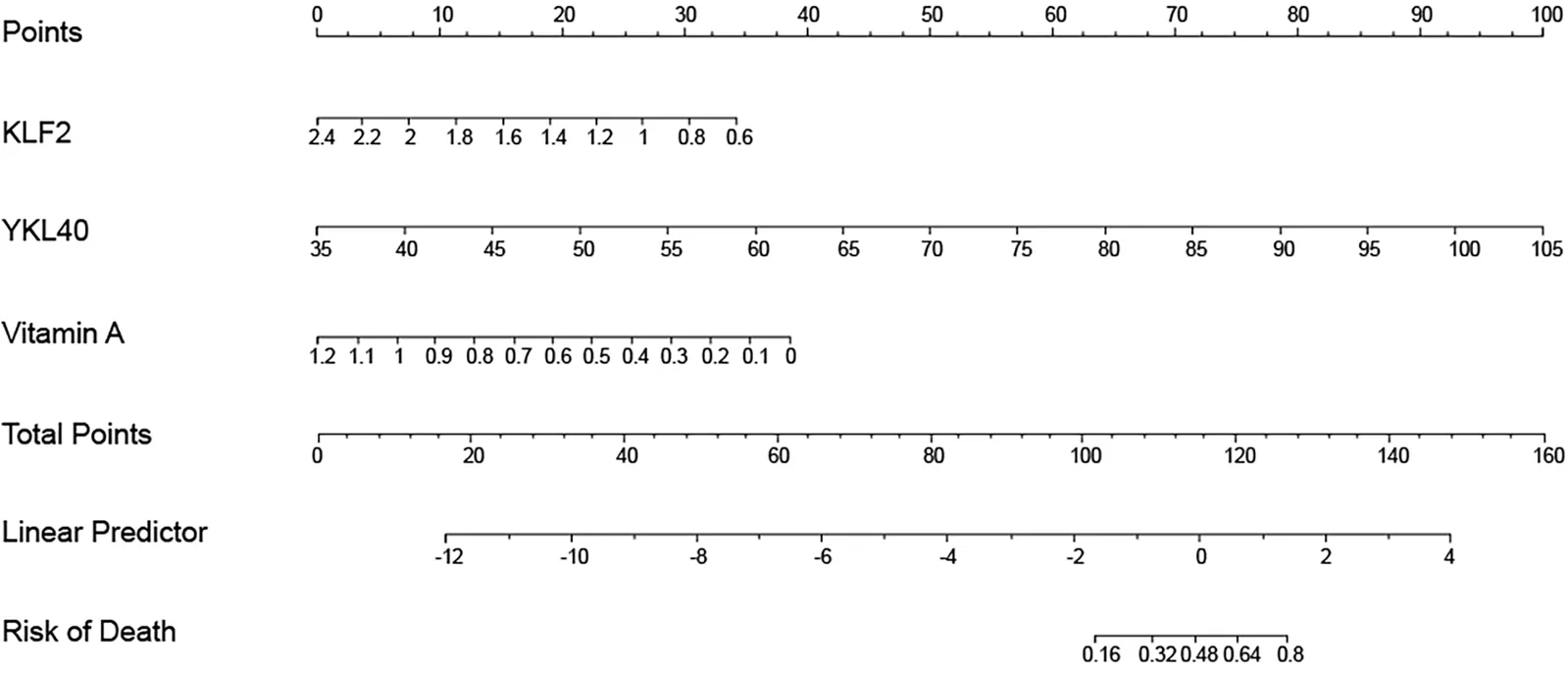
Nomogram for predicting poor prognosis of NRDS.

### Validation of the nomogram model

ROC analysis of the nomogram model affecting the prognosis of neonates with NRDS showed that its AUC = 0.918 (95% CI: 0.863–0.973), indicating good predictive ability (Figure 3A). The calibration curve is shown in Figure 3B. If the predicted value was between 15% and 20%, or 27%–32%, the calibration curve was located below, indicating overestimation of risk (true positive probability < predicted probability); in other cases, risk was basically underestimated (true positive probability > predicted probability). The Hosmer-Lemeshow goodness-of-fit test showed (*χ*^2^ = 8.210, *P* = 0.143); internal prediction of the model was performed using cross-validation, with the training set proportion set to 0.8, and a naive Bayes model with a Gaussian feature distribution was used for Bayesian modeling. The results showed that the final model achieved an accuracy of 87.50%, a precision (comprehensive) of 87.27%, a recall (comprehensive) of 87.50%, and an f1-score (comprehensive) of 0.87 on the test set. The model performance was acceptable. Validation of the predictive performance of the prediction model for the prognosis of neonates with NRDS using the DCA curve found that when the threshold probability was within the range of 0–0.88, the model had a positive net benefit (Figure 3C).

**Figure 3:**
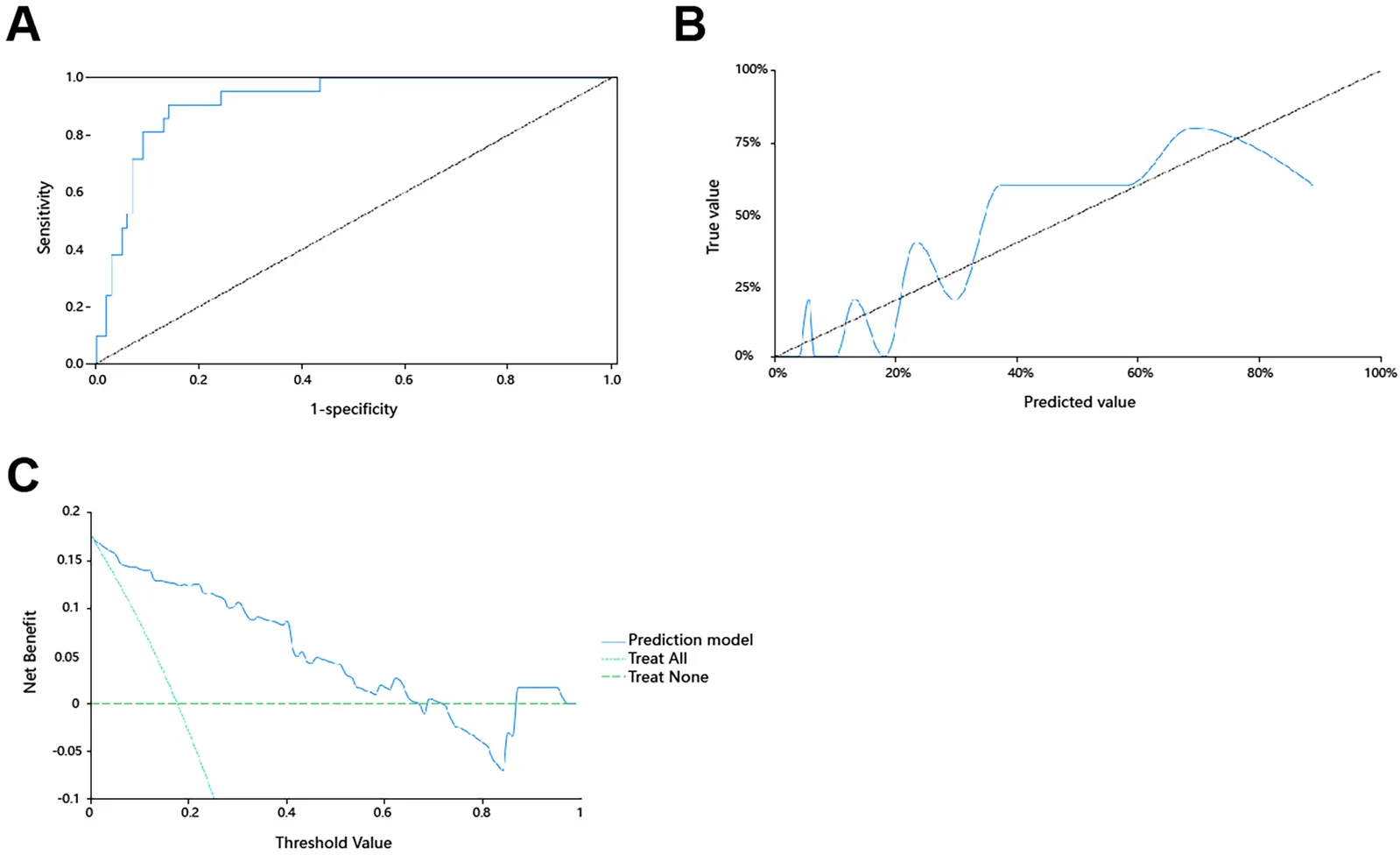
Validation of the nomogram model affecting poor prognosis of NRDS. (A) ROC curve (B) calibration curve (C) DCA curve.

## Discussion

The present study identified distinct alterations in serum KLF2, YKL-40, and vitamin A levels across different severities of NRDS. As disease severity increased, KLF2 and vitamin A levels gradually decreased, while YKL-40 levels rose. More importantly, each biomarker was associated with mortality, and their combined assessment achieved the highest predictive performance, with an AUC of 0.918. The present study systematically evaluated the predictive value of this biomarker panel for in-hospital mortality in infants with NRDS. This distinction has important clinical implications, as mortality risk stratification provides more direct guidance for clinical decision-making. This supports the novelty of our study.

Among the investigated biomarkers, KLF2 showed a clear inverse association with both disease severity and mortality. KLF2 is a key transcription factor involved in maintaining endothelial homeostasis and regulating inflammatory responses through suppression of adhesion molecules and pro-inflammatory mediators (17). Experimental studies have also implicated KLF2 in pulmonary development and protection against lung injury. Wang et al. reported that KLF2 expression was significantly reduced in neonatal rats with hyperoxia-induced lung injury, whereas restoration of KLF2 expression alleviated alveolar damage and attenuated inflammatory responses through inhibition of NF-*κ*B signaling (7). Furthermore, animal model studies have demonstrated that KLF2 inhibition is associated with impaired lung maturation, and KLF2 has been proposed to potentially participate in the differentiation of type I alveolar epithelial cells (18). A more recent study found that KLF2 expression is downregulated in neonates with persistent pulmonary hypertension of the newborn (PPHN), and KLF2 deficiency impairs pulmonary artery endothelial cell function and promotes pulmonary vascular remodeling (19). Since PPHN and NRDS frequently coexist and mutually exacerbate each other in clinical practice, this mechanistic link suggests that reduced KLF2 may aggravate NRDS severity by simultaneously affecting both the surfactant system and the pulmonary vascular system. Although experimental studies have implicated KLF2 in pulmonary injury and repair, clinical data in neonatal respiratory diseases remain scarce. The present findings suggest that reduced circulating KLF2 levels are associated with both greater disease severity and an increased risk of mortality in infants with NRDS. These observations support a potential role of KLF2 in the progression of neonatal lung injury and highlight its value as a candidate prognostic biomarker.

In contrast to KLF2, YKL-40 levels increased progressively with worsening disease severity and reached the highest concentrations in non-survivors. YKL-40 is secreted primarily by activated macrophages and neutrophils and has been implicated in inflammation, tissue remodeling, and fibrotic responses (20). A previous study demonstrated that YKL-40 expression is elevated during acute lung injury and contributes to inflammatory amplification and cellular apoptosis (11). Experimental suppression of YKL-40 has been shown to reduce inflammatory injury and improve cellular survival (21). More recently, elevated serum YKL-40 levels were reported in infants with NRDS and were associated with an increased risk of mortality (15). Consistent with these observations, our results further support the prognostic significance of YKL-40, whose standalone predictive performance was the highest among the three markers (AUC = 0.848), suggesting that persistent inflammatory activation may be one of the core drivers of poor prognosis in NRDS.

Vitamin A exhibited a pattern similar to that of KLF2, with progressively lower concentrations observed in more severe disease and among non-survivors. Vitamin A plays an essential role in lung development, epithelial integrity, and tissue repair. Experimental evidence indicates that vitamin A deficiency aggravates hyperoxia-induced lung injury by disrupting oxidative stress regulation and metabolic homeostasis (22). In addition, a recent meta-analysis demonstrated that vitamin A supplementation reduced the incidence of bronchopulmonary dysplasia in very preterm infants (22). Mechanistic studies have further revealed that retinoic acid, the active metabolite of vitamin A, directly upregulates the expression of surfactant protein B (SP-B) via the retinoic acid receptor (RAR) (23). Animal experiments have confirmed that vitamin A deficiency leads to a 21% reduction in total pulmonary surfactant phospholipids and a 32% decrease in SP-B mRNA levels in fetal rats (24). Recent research has provided reverse evidence by demonstrating that blockade of retinoic acid signaling with the RAR antagonist BMS493 significantly inhibits the transdifferentiation of alveolar epithelial cells (25). These findings indicate that vitamin A deficiency may accompany disease progression and could contribute to impaired pulmonary recovery in infants with NRDS.

The present study observed that changes in KLF2, YKL-40, and vitamin A levels may reflect the pathophysiological process of NRDS from different perspectives. The pathogenesis of NRDS primarily involves two mechanisms: Gestational diabetes mellitus (GDM)-induced delayed lung maturation and genetic defects leading to surfactant deficiency. The hyperglycemic environment in GDM antagonizes the promoting effect of glucocorticoids on surfactant synthesis through fetal hyperinsulinemia, downregulating SP-B and SP-C expression and exacerbating lung injury (15); meanwhile, GDM-related oxidative stress may affect KLF2 expression via the AMPK pathway. In terms of genetic factors, mutations in surfactant-related genes such as SP-B, SP-C, and ABCA3 are important causes of congenital surfactant deficiency, among which ABCA3 mutations are associated with severe NRDS phenotypes and poor response to conventional surfactant replacement therapy (26, 27). Vitamin A regulates SP-B transcription through the RAR pathway, suggesting that reduced vitamin A levels may further aggravate hereditary surfactant dysfunction (15). However, the present study did not include data on maternal glycemic control, placental pathology, or genetic sequencing, and the above associations await further validation through basic experiments and genetic analyses.

A nomogram model for the risk of poor prognosis in neonates with NRDS was constructed using KLF2, YKL-40, and vitamin A as predictors, with a total score of 0–160 corresponding to a probability of 0.16–0.80; the higher the patient's score, the higher the risk of poor prognosis. The model showed good predictive ability (AUC = 0.918, 95% CI: 0.863–0.973), the calibration curve indicated good fit (Hosmer-Lemeshow test *P* = 0.143), the internal validation accuracy was 87.50%, and the DCA curve showed that the model had a positive net benefit across a threshold probability range of 0–0.88. These results suggest that the nomogram model constructed from the three serum markers combined has good predictive performance and clinical net benefit for poor prognosis in children with NRDS. The reason the combined model outperforms any single marker may be attributable to the different biological processes represented by the three biomarkers: KLF2 is related to pulmonary endothelial and epithelial homeostasis, YKL-40 reflects inflammatory activity, and vitamin A participates in lung maturation and repair. Therefore, simultaneous assessment of these pathways may better represent the disease state than any single marker, which is also supported by the significantly improved predictive accuracy of the combined model. This model may serve as a potential tool for early identification of high-risk NRDS children and for assisting clinical decision-making; however, only internal validation has been completed so far, and it has not yet been validated in external multicenter cohorts, so its value for clinical dissemination still requires further prospective studies for confirmation.

At the level of clinical decision-making, the potential application value of these markers can be reflected in the following aspects. First, early risk stratification and escalation of intervention: for children whose early admission testing indicates low KLF2, high YKL-40, and low vitamin A, consideration may be given to earlier escalation of respiratory support, more active use of surfactants, and closer monitoring of blood gases and inflammatory indicators, rather than waiting for conventional indicators to worsen before intervening. Second, dynamic monitoring of treatment response: if serial monitoring shows persistently elevated YKL-40 or continuously declining KLF2 and vitamin A, this may indicate poor treatment response, and consideration should be given to adjusting the ventilation mode, strengthening anti-inflammatory treatment, or evaluating the benefit of vitamin A supplementation. Third, the modifiability of vitamin A deficiency: low vitamin A levels are associated with non-survivors, and vitamin A directly regulates SP-B transcription through the RAR pathway, suggesting that vitamin A supplementation in NRDS children with low vitamin A levels may not only improve nutritional status but also improve lung function by upregulating surfactant protein expression; however, the current study is observational in design, the direction of causality remains unclear, and prospective interventional trials are needed for verification. Fourth, an auxiliary basis for de-escalation decisions: for children whose three markers are all within the low-risk range, this may support earlier lowering of the level of monitoring and reduction of invasive procedures, thereby optimizing the allocation of medical resources.

This study has several limitations. First, it is a single-center, retrospective study, with samples from only one hospital, and it relies on existing medical records; both the study subjects and variable information were obtained retrospectively, so selection bias cannot be completely avoided, the sample size is relatively limited, and the generalizability of the results may be restricted. Second, some important clinical variables that may affect the outcome of death (such as gestational age, birth weight, prenatal glucocorticoid exposure, and disease severity) were not included in the multivariate model, which may constitute residual confounding; at the same time, no subgroup analysis was performed in this study, so it cannot be assessed whether the predictive performance of the markers is consistent across different subgroups. Third, the biomarkers were measured only once within 24 h after diagnosis, which cannot reflect their temporal trends or their relationship with prognostic evolution; serial measurements may further improve prognostic accuracy and are worth exploring in future studies. Fourth, no mechanistic experiments were conducted in this study, and the biological pathways linking changes in KLF2, YKL-40, and vitamin A to adverse outcomes remain incompletely understood. Finally, this combined biomarker model has not yet been validated in an external independent cohort, and its stability and generalizability still require further evaluation.

In summary, serum KLF2, YKL-40, and vitamin A levels were closely associated with both disease severity and prognosis in infants with NRDS. Combined assessment of these biomarkers provided superior predictive performance for mortality compared with individual markers alone. These findings suggest that a multi-biomarker approach may facilitate early risk assessment and prognostic evaluation in infants with NRDS. In response to the limitations of this study, future research can be carried out in the following directions: First, conduct multicenter, prospective cohort studies, expand the sources of samples, systematically collect variables such as gestational age, birth weight, prenatal glucocorticoid exposure, and disease severity, fully adjust for confounding factors, and perform subgroup analyses. Second, perform repeated sampling and dynamic monitoring of biomarkers to clarify the optimal detection time window and their relationship with prognostic evolution. Third, combine basic experiments to explore the specific mechanisms by which KLF2, YKL-40, and vitamin A participate in the progression of NRDS. Fourth, verify the stability and generalizability of the model through bootstrap resampling, cross-validation, and external independent cohort validation, and explore the feasibility of translating it into clinical decision-making.

## Data Availability

The original contributions presented in the study are included in the article/Supplementary Material, further inquiries can be directed to the corresponding author.
