# Supplementary material for: Serum KLF2, YKL-40, and vitamin A levels and their prognostic value in neonatal respiratory distress syndrome
Source: Front Pediatr. 2026 Oct 29;14:1898953. doi: 10.3389/fped.2026.1898953 (PMC13755317; doi:10.3389/fped.2026.1898953)
Supplement: Supplementary file 1 [file Table1.docx]

**Supplementary Table 1** Collinearity statistics

|  | Unstandardized coefficients | | Standardized coefficients | t | *P* | Collinearity statistics | |
| --- | --- | --- | --- | --- | --- | --- | --- |
|  | B | Standard Error | Beta |  |  | Tolerance | VIF |
| (Constant) | 0.001 | 0.32 |  | 0.003 | 0.998 |  |  |
| KLF2 | -0.301 | 0.108 | -0.238 | -2.796 | 0.006 | 0.845 | 1.184 |
| YKL40 | 0.011 | 0.003 | 0.335 | 3.902 | 0 | 0.833 | 1.201 |
| vitaminA | -0.23 | 0.129 | -0.146 | -1.789 | 0.076 | 0.926 | 1.079 |
